# Supplementary material for: PK Modeling of L-4-Boronophenylalanine and Development of Bayesian Predictive Platform for L-4-Boronophenylalanine PKs for Boron Neutron Capture Therapy
Source: Pharmaceuticals (Basel). 2024 Feb 26;17(3):301. doi: 10.3390/ph17030301 (PMC10975701; doi:10.3390/ph17030301)
Supplement: Supplementary file 1 [file pharmaceuticals-17-00301-s001.zip › pharmaceuticals-2736774-supplementary.pdf]

Supplementary Files

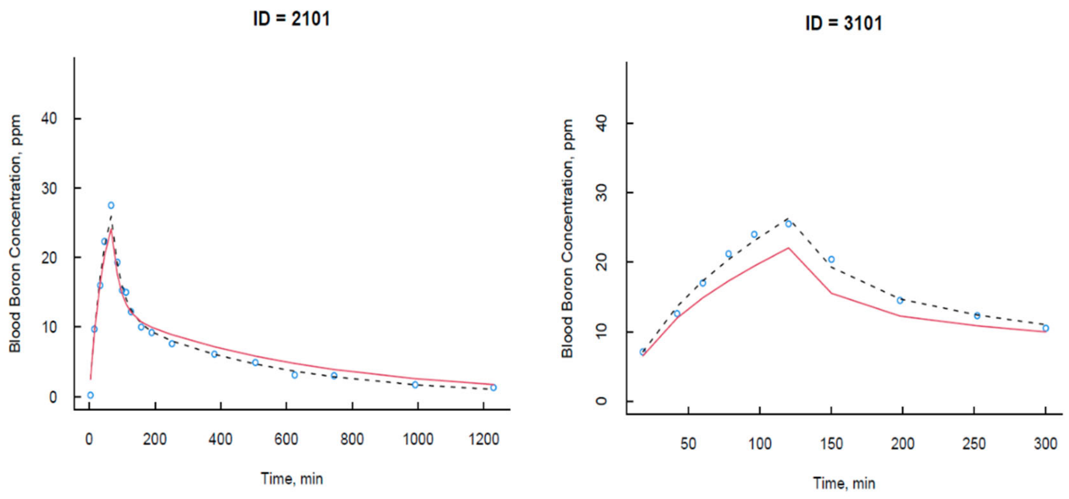

Figure S1. An example of individual prediction and population mean based on individual sample data in the literature. The red line indicates the population mean and the dotted line indicates the individual prediction using the constructed pharmacokinetic model showing high accuracy.

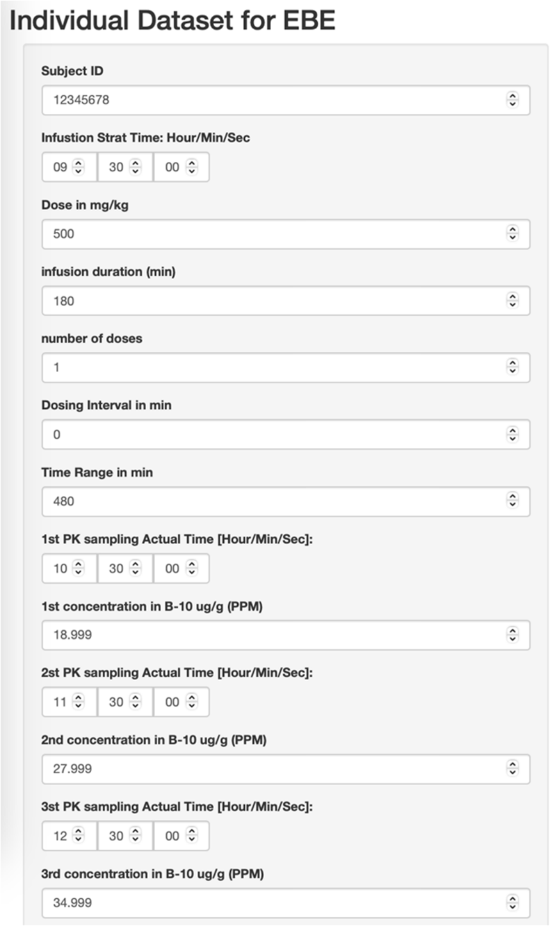

ID	TIME	AMT	RATE	DV	MDV	ADDL	II	WT
12345678	0	.	.	.	1	.	.	60
12345678	0	500	0.13290802764487	.	1	0	0	60
12345678	0.48048048048048	.	.	.	1	.	.	60
12345678	0.960960960960961	.	.	.	1	.	.	60
12345678	1.44144144144144	.	.	.	1	.	.	60
12345678	1.92192192192192	.	.	.	1	.	.	60
12345678	2.4024024024024	.	.	.	1	.	.	60
12345678	2.88288288288288	.	.	.	1	.	.	60
12345678	3.36336336336336	.	.	.	1	.	.	60
12345678	3.84384384384384	.	.	.	1	.	.	60
12345678	4.32432432432432	.	.	.	1	.	.	60
12345678	4.8048048048048	.	.	.	1	.	.	60
12345678	5.28528528528529	.	.	.	1	.	.	60
12345678	5.76576576576577	.	.	.	1	.	.	60
12345678	6.24624624624625	.	.	.	1	.	.	60
12345678	6.72672672672673	.	.	.	1	.	.	60
12345678	7.20720720720721	.	.	.	1	.	.	60
12345678	7.68768768768769	.	.	.	1	.	.	60
12345678	8.16816816816817	.	.	.	1	.	.	60
12345678	8.64864864864865	.	.	.	1	.	.	60
12345678	9.12912912912913	.	.	.	1	.	.	60
12345678	9.60960960960961	.	.	.	1	.	.	60
12345678	10.0900900900901	.	.	.	1	.	.	60
12345678	10.5705705705706	.	.	.	1	.	.	60
12345678	11.051051051051	.	.	.	1	.	.	60
12345678	11.5315315315315	.	.	.	1	.	.	60
12345678	12.012012012012	.	.	.	1	.	.	60
12345678	12.4924924924925	.	.	.	1	.	.	60
12345678	12.972972972973	.	.	.	1	.	.	60
12345678	13.4534534534535	.	.	.	1	.	.	60

Figure S2. Graphical User Interface (GUI) of the Rshiny Platform for Individual Dataset for Event-Based Estimations (EBE). The left section of the GUI is designed for user input, where essential details such as Subject ID, Infusion Start Time, Dose, Infusion Duration, Number of Doses, Dosing Interval, Time Range, and specific timepoints for PK sampling and corresponding B-10 concentrations can be entered. The right section displays a generated dataset reflecting the time course of B-10 concentration levels post-infusion. This systematic layout ensures a streamlined workflow from data entry to the visualization of individualized pharmacokinetic profiles, facilitating the estimation of B-10 concentration based on real-time patient data.
